# Health services research in the public healthcare system in Hong Kong: An analysis of over 1 million antihypertensive prescriptions between 2004–2007 as an example of the potential and pitfalls of using routinely collected electronic patient data

**DOI:** 10.1186/1472-6963-8-138

**Published:** 2008-06-25

**Authors:** Martin CS Wong, Johnny Y Jiang, Jin-ling Tang, Augustine Lam, Hong Fung, Stewart W Mercer

**Affiliations:** 1 Department of Community and Family Medicine and School of Public Health, Chinese University of Hong Kong, Hong Kong SAR, China. 4/F, School of Public Health, Prince of Wales Hospital, Shatin, HKSAR, PR China; 2 General Practice and Primary Care, Division of Community-Based Sciences, Faculty of Medicine, University of Glasgow, 1 Horselethill Road, Glasgow G12 9LX, UK

## Abstract

**Background:**

Increasing use is being made of routinely collected electronic patient data in health services research. The aim of the present study was to evaluate the potential usefulness of a comprehensive database used routinely in the public healthcare system in Hong Kong, using antihypertensive drug prescriptions in primary care as an example.

**Methods:**

Data on antihypertensive drug prescriptions were retrieved from the electronic Clinical Management System (e-CMS) of all primary care clinics run by the Health Authority (HA) in the New Territory East (NTE) cluster of Hong Kong between January 2004 and June 2007. Information was also retrieved on patients' demographic and socioeconomic characteristics, visit type (new or follow-up), and relevant diseases (International Classification of Primary Care, ICPC codes).

**Results:**

1,096,282 visit episodes were accessed, representing 93,450 patients. Patients' demographic and socio-economic details were recorded in all cases. Prescription details for anti-hypertensive drugs were missing in only 18 patients (0.02%). However, ICPC-code was missing for 36,409 patients (39%). Significant independent predictors of whether disease codes were applied included patient age ≥ 70 years (OR 2.18), female gender (OR 1.20), district of residence (range of ORs in more rural districts; 0.32–0.41), type of clinic (OR in Family Medicine Specialist Clinics; 1.45) and type of visit (OR follow-up visit; 2.39).

In the 57,041 patients with an ICPC-code, uncomplicated hypertension (ICPC K86) was recorded in 45,859 patients (82.1%). The characteristics of these patients were very similar to those of the non-coded group, suggesting that most non-coded patients on antihypertensive drugs are likely to have uncomplicated hypertension.

**Conclusion:**

The e-CMS database of the HA in Hong Kong varies in quality in terms of recorded information. Potential future health services research using demographic and prescription information is highly feasible but for disease-specific research dependant on ICPC codes some caution is warranted. In the case of uncomplicated hypertension, future research on pharmaco-epidemiology (such as prescription patterns) and clinical issues (such as side-effects of medications on metabolic parameters) seems feasible given the large size of the data set and the comparability of coded and non-coded patients.

## Background

The use of electronic patient records within healthcare systems has important implications for health services research. When used within a comprehensive computerized data management system such records can support research into the aetiology of disease [1,2], the predictive value of symptoms in diagnosis [3], the clinical effectiveness and cost effectiveness of interventions [4], and in evaluating whole-system approaches to the organization and delivery of care [5]. The databases invariably contain a large number of subjects, may provide almost complete population coverage (depending on the healthcare and data collection systems), often provide reliable information on variables such as demographic characteristics, prescribing patterns and diagnoses, and in theory allow for quick and efficient data retrieval [6]. Notable examples include the UK General Practice Research Database (GPRD) [7], the Health Search Database in Italy [8], and Ontario Drug Benefit (ODB) database [9]. Some databases include almost all residents of a province [10,11], thus enhancing the applicability and generalizability of findings.

In many western countries these databases have been constructed in a systematic manner and influential study results have been published [12-15]. There is a growing trend for these evolving information systems to implement regional networks [16] and allow physician- or patient- access to clinical information, as well as integration of a broader spectrum of patient data. Hong Kong initiated the operation of a comprehensive computerized recording and management system in the public sector in 2000. All patient information, drug prescription details and laboratory investigation results are routinely entered into the electronic Clinical Management System (e-CMS) for every consultation by health care professionals, backed up by the Clinical Data Analysis Reporting System (CDARS). Paper records will only be used during rare occasions where computer systems are unexpectedly not operational. These computerized databases thus far consist of seven million patient records, one million annual admissions and 13 million ambulatory visits, with medical research as one of the stated purposes of their implementation [17]. Nevertheless, the usefulness of these clinical electronic databases in Hong Kong for high quality health services research has been little explored [17].

Hypertension is a good example of a common and important condition which can be successfully researched using comprehensive computerized patient records [8,18-21]. The prevalence of hypertension in Hong Kong has been reported to exceed 27% and is one of the most common conditions seen in public health sector [22], comparable to the worldwide figure of 26% [23]. The public health costs of hypertension are substantial [24,25] and represent an important area of health service research [26-30], but there is a paucity of reports on health care utilization patterns of this disease among Chinese patients. In the present study we evaluated the completeness of the demographic and prescription details of patients recorded in the e-CMS database of the HA in Hong Kong. Also we examined the patterns and independent predictors of disease coding using antihypertensive drug prescriptions in primary care as an example.

## Methods

### Data Source and Subjects

Clinical and demographic information were retrieved from CDARS for all patients who attended HA primary care clinics in the New Territory East (NTE) cluster of Hong Kong, during the period from January 2004 to June 2007. We chose this time period because although e-CMS was initiated in 2000, its implementation was gradual and was severely interrupted by the SARS outbreak in 2003. There was no requirement for doctors to enter the ICPC codes into the CMS until post-SARS, which is also when CDARS became operationalised. Thus January 2004 is the earliest time point for the availability of both CDARS and e-CMS data including ICPC codes.

The NTE cluster serves a population of around 1.3 million in Hong Kong, representing 17.2% of the Hong Kong population [31]. This cluster is further divided into 3 separate regions, namely Shatin, Tai-Po and the North District, from the most urbanized to the most rural regions respectively. Their median monthly household incomes in 2006 were US$2,510, US$2,338 and US$2,078 for these three regions respectively, compared to the Hong Kong-wide figure of US$2,240 [31]. These three regions have similar median ages (38–39 years), comparable with the median age of 39 years for Hong Kong.

Eligible subjects were those having received at least one prescription of antihypertensive drugs in any of these primary care visits. Only visits which result in prescription of antihypertensive drugs were included (hereafter named "antihypertensive drug visits"). Since CDARS does not have disease coding, we further obtained a list of patient identity card (ID) numbers from CMS for various relevant codes of the International Classification of Primary Care (ICPC), including uncomplicated hypertension (K86) and other conditions which could potentially affect the prescription choice of antihypertensive drugs, which we therefore refer to as "exclusion codes" (Table 1). These codes were then merged with the information from CDARS using the patients' ID as the unique identifier. Patients were considered 'coded' if they had at least one relevant ICPC code entered in the CMS during the study period. The study was approved by the NTE cluster of HA, and the Survey and Behavioural Research Ethics Committee, Chinese University of Hong Kong

**Table 1:** Distribution of International Classification of Primary Care (ICPC) codes among patients with ≥ 1 code (N = 57,041)

**ICPC codes**	**Disease entity**	**Reasons for exclusion**	**number**	**%**
K87	Complicated hypertension	The presence of unknown complications may favor or preclude prescription of a particular drug class	3310	5.8%
T90	Diabetes Mellitus	Favors the choice of ACEIs	15560	27.3%
T901	Impaired glucose tolerance		353	0.6%
T92	Gout	Contraindication of thiazide diuretics	635	1.1%
T93	Lipid disorders	Favor the exclusion of thiazide diuretics and β-blockers	1186	2.1%
K90	Stroke/cerebrovascular accident	Favor the choice of β-blockers	1920	3.4%
K91	Cerebrovascular disease		130	0.2%
K74	Ischemic Heart Disease with angina		348	0.6%
K76	Ischemic Heart Disease without angina		1099	1.9%
K75	Acute Myocardial Infarction		213	0.4%
K77	Heart Failure	Favor the choice of ACEIs	794	1.4%
K84	Heart Disease, other	Favor the choice of β-blockers or ACEIs	168	0.3%
K99	Cardiovascular disease, other		113	0.2%
R79	Chronic Bronchitis	Contraindication of β-blockers	436	0.8%
R95	Chronic Obstructive Pulmonary Disease		2199	3.9%
R96	Asthma		1083	1.9%
U14	Kidney symptoms/complaints	Either favor or a contraindication of ACEIs	312	0.5%
U88	Glomerulonephritis/nephrosis		36	0.1%
Y85	Benign Prostatic Hypertrophy	Favor the prescription of α-blockers	2917	5.1%
U78	Benign Neoplasm Urinary Tract		30	0.1%
Y79	Benign/unspecified neoplasm, male genital		1	0.0%

(ACEIs: Angiotensin Converting Enzyme Inhibitors. There were a total of 46,859 patients (82.1%) Apart from K86 these conditions are influencing factors for antihypertensive drug prescription and were named "exclusion codes"

### Variables and Statistical Analysis

All retrieved data were transformed and analyzed by the Statistical Package for Social Sciences (SPSS) version 13.0. The proportions of four distinct, mutually exclusive patient groups were reported. These consist of (1). Those coded with K86 and with exclusion coding ("combined group"); (2). Those coded with K86 without any exclusion coding ("K86-only group"); (3). Those not coded with K86 but with at least one exclusion coding ("Exclusion only group"); and (4). Those without K86 nor any exclusion coding ("Non-coded group"). Among patients with at least one code, the distribution of each coding was studied. The basic demographic and health service characteristics of these different coding groups were compared using χ^2 ^tests of homogeneity and Analysis of Variance (ANOVA) for categorical and continuous variables, respectively. These include patient age, gender, payment status (fee-waivers or service payers), district of residence, service type (general out-patient, Family Medicine Specialist Clinic (FMSC), staff clinics) and appointment type (new or subsequent visits) All these data were routinely entered by clinic staffs when patients attended for consultation, apart from service type. We further analyzed the independent predictors of patients having received at least one ICPC code, as those listed in Table 1, by physicians using bivariate analysis, followed by multivariable analysis by entering predictors with statistically significance into a binary logistic regression equation. A forward stepwise model was adopted and only those significant independent predictors were reported. All p values <0.05 were regarded as statistically significant.

## Results

### Overall patient breakdown

**Figure 1:**
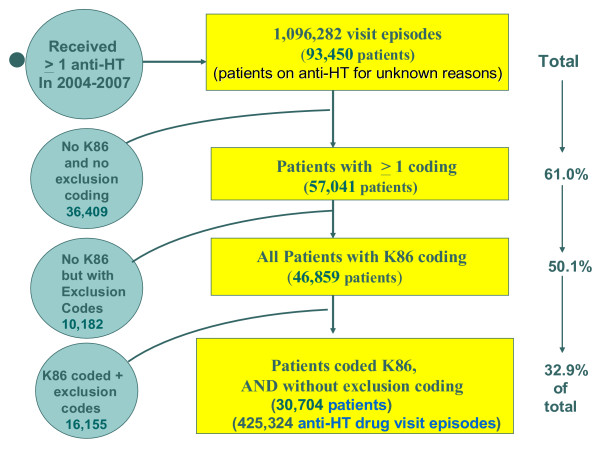
The breakdown of disease coding relevant to antihypertensive (anti-HT) drug prescriptions.

A total of 1,096,282 antihypertensive drug visits were retrieved from January 2004 to June, 2007, representing 93,450 patients. Demographic and socio-economic details were recorded for all patients. Prescription details for anti-hypertensive drugs were missing in only 18 patients (0.02%), including drug dosage, frequency, or prescription period, and a combination of these. Figure 1 shows the breakdown of disease coding relevant to antihypertensive (anti-HT) drug prescriptions. Relevant ICPC-codes were absent in 36,409 patients (39% of the total sample); i.e., these patients had neither K86 nor any exclusion code. The remaining 57,041 (61% of total) patients had at least one relevant disease identified by an ICPC code. Of these, 46,859 (50.1% of the total sample) had uncomplicated hypertension (K86) coding, and among these K86-coded patients, 16,155 patients (17.2%) also had at least one additional (exclusion) code, resulting in 30,704 patients (32.9% of total) with uncomplicated hypertension (K86) without associated co-morbidity (i.e., no exclusion codes).

Most of the patients were receiving calcium channel blockers (49%) and β-blockers (46%), followed by angiotensin converting enzyme inhibitors (19%) and thiazide diuretics (13%).

### Distribution of codes and morbidities

Out of the 57,041 patients (61.0%) with at least one ICPC disease code, the majority (82.1%) had uncomplicated hypertension (K86) followed by diabetes mellitus (T90, 27.3%), complicated hypertension (K87, 5.8%) and benign prostatic hypertrophy (Y85, 5.1%) (Table 1). The coding proportions of other relevant diseases were low. Among patients with at least one coded condition, 66.7% had one code (or one condition) and 27.8% two or more from the list of conditions shown in Table 1. Among patients coded with K86, the figures are similar (65.5% K86 only and 29.5% K86 coded with exclusion codes, respectively).

### Comparing patient characteristics among different coding groups

The whole study cohort had an average age of 64 years, with the majority (61.2%) being 60 years old or over (Table 2). Patients with uncomplicated hypertension (K86-only group) had a similar age distribution to the non-coded group. Overall there were more female than male patients (Table 2). The K86-only and non-coded groups had more female patients than the other two groups. Almost half of all patients lived in the Shatin region, which is the most urbanized area in the NTE cluster (Table 2). More patients from the K86-only group lived in the Shatin district than the other groups. The non-coded group had the smallest proportion of patients residing in Shatin (33%).

**Table 2:** Patient characteristics according to K86 & exclusion coding

**Coding**	**K86 coded & without exclusion codes (n = 30,704)**	**K86 coded with exclusion codes (n = 16,155)**	**No K86 but with exclusion codes (n = 10,182)**	**No K86 & no exclusion codes (36,409)**	**All cases (n = 93,450)**	p value*
**Patient**	"K86 only group"	"combined group"	"Exclusion group"	"Non-coded group"		

***Age on appt. (no.%)***						
<50	4550(14.8)	1,073 (6.6)	791 (7.8)	7,484 (20.6)	13898(14.9)	<0.001
50–59	8116(26.4)	2,814 (17.4)	1,804 (17.7)	9,704 (26.7)	22438(24.0)	
60–69	6954(22.6)	4,191(25.9)	2,421 (23.8)	7,516 (20.6)	21082(22.6)	
≥70	11084(36.1)	8,077 (50.0)	5,166 (50.7)	11,705 (32.1)	36032(38.6)	
***Mean Age on appt*. **(SD) (95% C.I.)	63.87(13.06)	68.22 (11.61)	68.45 (12.46)	61.77 (14.26)	64.30 (13.51)	<0.001
	(63.73, 64.02)	(68.04, 68.40)	(68.21, 68.69)	(61.62, 61.91)	(64.21, 64.39)	
***Gender ***(No./**%**)						
*Male*	11,323 (36.9)	7,442 (46.1)	6,153 (60.4)	15,412 (42.3)	40330 (43.2)	<0.001
*Female*	19,381 (63.1)	8,713 (53.9)	4,029 (39.6)	20,997 (57.7)	53120 (56.8)	
***Payment status***						
Fee-waivers	7,949 (25.9)	4,379 (27.1)	3,147 (30.9)	9,696 (26.6)	25,171 (26.9)	<0.001
Non- waivers	22,755 (74.1)	11,776 (72.9)	7,035 (69.1)	26,713 (73.4)	68,279 (73.1)	
***District of residence***						
Shatin	18,510 (60.3)	8,817 (54.6)	5,524 (54.3)	12,120 (33.3)	44,971 (48.1)	<0.001
Taipo	6,780 (22.1)	2,883 (17.8)	1,773 (17.4)	11,156 (30.6)	22,592 (24.2)	
North	4,068 (13.2)	3,721 (23.0)	2,206 (21.7)	10,684 (29.3)	20,679 (22.1)	
Others	1,346 (4.4)	734 (4.5)	679 (6.7)	2,449 (6.7)	5,208 (5.6)	
***Service type***						
General	28,946 (94.3)	13,881 (85.9)	7,523 (73.9)	34,524 (94.8)	84,874 (90.8)	<0.001
FMSC/IC	1,625 (5.3)	2,260 (14.0)	2,618 (25.7)	1,673 (4.6)	8,176 (8.7)	
Staff clinic	133 (0.4)	14 (0.1)	41 (0.4)	212 (0.6)	400 (0.4)	
***Appointment type (no./%)***						
New case	14,705 (47.9)	5,485 (34.0)	4,784 (47.0)	23,831 (65.5)	48,805 (52.2)	<0.001
Old case	15,999 (52.1)	10,670 (66.0)	5,398 (53.0)	12,578 (34.5)	44,645 (47.8)	

(*: Pearson Chi Square tests)

Most patients attended the primary care general out-patient clinics for antihypertensive drugs, but the exclusion group had more patients who attended FMSC. The distribution of service types was similar between the K86-only group and the non-coded group. Overall more than half of the patients were 'new' in terms of antihypertensive drug visits during the study period. The non-coded group had the greatest proportion of new visits (66%), almost twice as high as in the combined group (34.0%).

### Factors associated with disease coding

Multi-regression logistic regression was used to determine the independent predictors of disease coding, i.e., the factors associated with whether a patient receiving antihypertensive drugs had an ICPC disease code or not. Younger patients (< 50 years) were less likely to be coded than older patients (Table 3). Female patients were more likely to be coded (aOR = 1.202, 95% C.I. 1.168, 1.238, p < 0.001). Patients living in Shatin district were more likely to receive a code when compared to other less urbanized regions (aOR range from 0.316 to 0.405). Patients visiting family medicine specialist clinics were more likely to be coded when compared to general out-patient clinics (aOR = 1.448, 95% C.I. 1.362, 1.539, p < 0.001). Follow-up cases were more likely to have a code (aOR = 2.394, 95% C.I. 2.324, 2.467, p < 0.001). No significant association was found between payment status and physician coding.

**Table 3:** Associated factors of coding by physicians

**Coding**	Coded* (n = 57,041)	**Non-coded(n = 36,409)**	Adjusted Odds Ratios** (95% C.I.)	**p value**
**Patient**				
***Age on appt. (no.%)***				
<50	6,414(11.2)	7,484(20.6)	1.000 (reference)	
50–59	12,735(22.3)	9,704(26.7)	1.364(1.302, 1.429)	<0.001
60–69	13,566(23.8)	7,516(20.6)	1.774(1.692, 1.861)	<0.001
≥70	24,327(42.6)	11,705(32.1)	2.180(2.088, 2.277)	<0.001
***Gender***				
*Male*	24,918(43.7)	15,412(42.3)	1.000 (reference)	
*Female*	32,123(56.3)	20,997(57.7)	1.202(1.168,1.238)	<0.001
***Payment status***				
Fee-waivers	15,475(27.1)	9,696(26.6)		
Payers	41,566(72.9)	26,713(73.4)	Not significant	
***District of residence***				
Shatin	32,851(57.6)	12,120(33.3)	1.000 (reference)	
Taipo	11,436(20.0)	11,156(30.6)	0.316(0.304, 0.327)	<0.001
North	9,995(17.5)	10,684(29.3)	0.330(0.318, 0.343)	<0.001
Others	2,759(4.8)	2,449(6.7)	0.405(0.381, 0.432)	<0.001
***Service type***				
General	50,350(88.3)	34,524(94.8)	1.000 (reference)	
FMSC	6,503(11.4)	1,673(4.6)	1.448(1.362, 1.539)	<0.001
Staff clinic	188(0.3)	212(0.6)	0.940(0.760, 1.162)	0.565
***Appointment type (no./%)***				
New case	24,974(43.8)	23,831(65.5)	1.000 (reference)	
Old case	32,067(56.2)	12,578(34.5)	2.394(2.324, 2.467)	<0.001

(FMSC: Family Medicine Specialist Clinic. *Coded group refers to patients having at least one code, either K86 or exclusion coding. **represent odds ratios after controlling for all the predictor variables)

## Discussion

### Main findings

The present study shows that the public clinical databases on antihypertensive drugs in Hong Kong are complete in demographic and socio-economic data, and 99.98% complete in prescription details. Among patients receiving antihypertensive prescription in the primary care system, the proportion having any relevant disease code was relatively modest (61.0%) when compared with other practices in Western countries. About one third (32.9%) of patients were identified as uncomplicated hypertensive (K86-only) patients with no exclusion conditions. There was similarity however between the K86-only group and non-coded groups in age and gender distribution, payment status as well as service types, suggesting that most non-coded patients probably had uncomplicated hypertension. The important independent positive predictors of having an ICPC code were advanced age, female gender, residence in urbanized district, service type being family medicine specialty, and follow-up case.

### Interpretation of findings

The completeness of the demographic, socio-economic, and prescribing details relate to the way in which this information is organized and documented within the e-CMS. Patient must register at first attendance and the demographic and socio-economic data is entered directly into the computer system by administrative staff in the reception office. Prescriptions can only be issued via the CMS, including private prescriptions. Thus we would expect such data to be complete. ICPC codes on the other hand are entered by the attending physicians during the consultation. Most primary care clinics are extremely busy (each doctor needs to handle 70–80 patients a day), and short consultations are the norm rather than the exception Thus it is perhaps unsurprising that ICPC codes were often not entered into the e-CMS. Audit work has shown that diagnoses are often entered into the hand written notes rather than given an ICPC code (MCS Wong, unpublished data).

The finding that older patients are more likely to be coded can perhaps be explained by the presence of more morbidities in the elderly group and perhaps more frequent consulting (and hence likelihood of ICPC code being entered on at least one occasion) which would also be the case for the higher coding rates in the follow-up patients. The coding difference in different districts of residence may possibly be explained by the concentration of training centres and teaching clinics in Shatin. The similarly higher coding rate in FMSC can also be explained by the more stringent coding requirement of specialty clinics. That higher likelihood of the female patients receiving a code is also of interest and further exploration of the reasons is needed.

Since completion of the current study, the NTEC, HA has reinforced its ICPC coding policy and the coding rate for all diseases as of July, 2007 has been reported to be 87.7% [information provided by statistics team of the Hospital Authority Head Office, November 2007]. Thus in future work using this database substantially higher percentages of disease coded data for patients with hypertension and other chronic conditions seems likely.

### Relationship to published literature

Many studies have used different diseases to validate local databases. For the GPRD database in the UK, hospital investigations and death certificates have been adopted to support the validity of disease codes for deep vein thrombosis and pulmonary embolism [32]. Questionnaires posted to general practitioners have been used to verify the diagnosis and severity of chronic obstructive pulmonary diseases for validation of the OXMIS codes of GPRD [33]. Both studies found good agreement between database disease codes and the tools used for validation, and concluded that GPRD is of sufficiently high quality for epidemiological research. Other methods for database validation include the use of morbidity surveys and national data to compare with database information [34,35], which reported similar database usefulness. Many studies on GPRD mainly focused on the completeness and quality of computer recorded data [36,37]. GPRD has been used to address a variety of clinical issues, including pharmaco-epidemiology and medication safety [38], rheumatoid diseases [39,40], gout [41,42], diabetes [43], and sexually transmitted infections [44], among others. The GPRD in UK consists of information from practices which are up to standard, requiring the practice to record a minimum of 95% of prescribing and relevant patient-encounter events [45]. Also data from practices are routinely under internal checks for validity [34], and each practice will be sent a validation report after data collection.

We are unaware of any published work on validation of the accuracy of disease codes in the HA e-CMS in Hong Kong, but clearly this would be an important area for future work if more rigorous health services research is planned. However, a first step is the need for implementation of a quality control system and the necessary organizational changes to ensure more complete data entry in terms of ICPC disease codes.

### Strengths and weaknesses of current study

The major strength of this study was the availability of a large amount of electronic patient data on antihypertensive medication over a three and a half year period from which we could ascertain the potential and pitfalls of using such data in health services research in the public healthcare system in Hong Kong. We have done this in a systematic way, and included robust statistical analysis to aid interpretation.

However, this study also has some weaknesses. Firstly, there are no external data to support the validity of the database. We regarded the ICPC code as the 'gold standard' in categorizing our study cohort. The accuracy of these codes however, has not been formally validated against data from case notes or those from the secondary care sector (which uses International Classification of Diseases (ICD) codes). However, given the relatively straightforward diagnosis of uncomplicated hypertension we believed the biases may be minimal. Secondly, these data apply only to the NTE cluster, which represents only one of seven organisational units within the HA in Hong Kong. Thus we cannot know if the issues raised in the study apply across the public healthcare system in Hong Kong, although we have no reason to suspect that NTE is different organizationally that other clusters. Thirdly, due to gradual implementation of the e-CMS system and the disruption of SARS in 2003 the present study could not evaluate ICPC coding before 2004, and some of the 'un-coded' patients in the present study could have been coded by physicians pre-2004 into hand-written records or free text space. However, since January 2004, all doctors in the clinics are required to use the computer system as the sole portal of information entry and patients with ICPC codes in hand-written notes or free text before January 2004 should have had that information re-entered into the e-CMS at consultations during the study period. However, the ICPC code need only be entered once to be counted as a 'coded patient' (not re-coded at every consultation).

### Implications for policy and practice

Hypertension is an important chronic disease globally and locally in Hong Kong. The large numbers of electronic patient records and completeness of some information could potentially serve to help addressing this condition in important areas of health services research, such as drug prescription profiles and health service utilization patterns. The scale of the database also has potential in clinical epidemiological research with linkage between the needs of the populations served, their geographical location, and the availability of high quality primary care on the basis of such needs. Data protection issues should however be observed by researchers; the present database is anonymized having only identity numbers without patient contact details hence enhancing its data safety. In practical terms, there are simple steps that can be initiated at minimal cost, to enhance the quality of the e-CMS data. For example, guidelines on ICPC coding for the clinical staff may be helpful, and system changes which require the entry of ICPC codes as compulsory rather than optional. Our finding that more family medicine specialist clinics are likely to have coding supports the effectiveness of routine data protocols in enhancing disease coding rates, because these clinics are doctor training centers where data recording protocols are more stringently managed and implemented. Further studies are needed to validate the accuracy and completeness of CDARS and CMS in Hong Kong by more rigorous testing. These include comparison of cluster-representative surveys with data generated for each disease entity to ensure they are representative, and exploration of the disease status of uncoded patients by case-note reviews to test whether the uncoded group is likely to have uncomplicated hypertension only.

Since prescription details are virtually complete, this database has the potential to study the patterns of antihypertensive prescription, profiles of drug discontinuation and switching, and the association of antihypertensive drug class to clinical outcomes like mortality. Work is underway currently on all these areas of enquiry.

## Conclusion

The e-CMS database of the HA in Hong Kong varies in quality in terms of recorded information. Potential future health services research using demographic and prescription information is highly feasible but for disease-specific research dependant on ICPC codes some caution is warranted. In the case of uncomplicated hypertension, future research on pharmaco-epidemiology (such as prescription patterns) and clinical issues (such as side-effects of medications on metabolic parameters) seems feasible given the large size of the data set and the comparability of coded and non-coded patients.

## Competing interests

The authors declare that they have no competing interests.

## Authors' contributions

MCSW has contributed to the conception, design, data acquisition, analysis of data and prepared the first draft of this paper. JYJ and J–LT contributed to refining the methodology, data analysis, and intellectual input to data interpretation; AL, HF and SWM contributed to amendment of initial study design, analysis of data, and critically revising the manuscript. All authors have read and approved the final manuscript. SWM is guarantor for the study.

## Pre-publication history

The pre-publication history for this paper can be accessed here:


